# Quantum Fourier transform to estimate drive cycles

**DOI:** 10.1038/s41598-021-04639-0

**Published:** 2022-01-13

**Authors:** Vinayak Dixit, Sisi Jian

**Affiliations:** 1 Research Centre for Integrated Transport Innovation (rCITI), School of Civil and Environmental Engineering, UNSW Sydney, Sydney, Australia; 2 Department of Civil and Environmental Engineering, The Hong Kong University of Science and Technology, Clear Water Bay, Kowloon, Hong Kong, SAR China

**Keywords:** Civil engineering, Mechanical engineering

## Abstract

Drive cycles in vehicle systems are important determinants for energy consumption, emissions, and safety. Estimating the frequency of the drive cycle quickly is important for control applications related to fuel efficiency, emission reduction and improving safety. Quantum computing has established the computational efficiency that can be gained. A drive cycle frequency estimation algorithm based on the quantum Fourier transform is exponentially faster than the classical Fourier transform. The algorithm is applied on real world data set. We evaluate the method using a quantum computing simulator, demonstrating remarkable consistency with the results from the classical Fourier transform. Current quantum computers are noisy, a simple method is proposed to mitigate the impact of the noise. The method is evaluated on a 15 qubit IBM-q quantum computer. The proposed method for a noisy quantum computer is still faster than the classical Fourier transform.

## Introduction

Drive cycles are important determinants of emissions, energy consumption and safety. Higher frequencies of acceleration and deceleration cycles are predictors of higher emissions, fuel consumption^1^ and crashes^2,3^. Regulators, vehicle manufacturers and traffic engineers are extremely interested to understand the driving cycles for implementing policies and real-time control systems.

**Figure 1:**
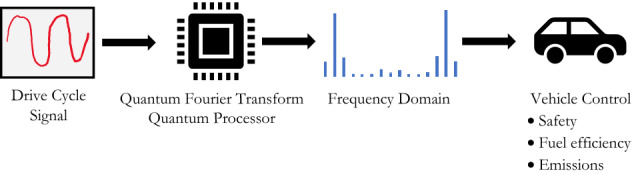
Quantum Fourier transform for drive cycle analysis.

Current vehicle systems rely on Fourier Transformation to evaluate the frequency domain for vehicle control. This study demonstrates the use of a Quantum Fourier Transform (QFT) to extract the frequency domain, which could have significant implications on ability to react faster to improve safety, fuel efficiency and reduce emissions (Fig. 1). QFT is a linear transformation of the amplitudes in the superposition of the qubits and is a key part of quantum algorithms^4–6^.

Research in quantum computing and algorithms over the past three decades have theoretically demonstrated the potential gains through “quantum speedup”^7^. At a fundamental level, quantum computers differ from classical computers in their ability to leverage quantum mechanical properties such as superposition, entanglement and interference to speedup computations.

Applications of quantum algorithms in the field of transportation and traffic have been limited. Bernas and Wisniewska^8^, provided a preliminary framework for expressing a Cellular Automata Traffic Model in a Quantum Computable Format. Though the initial approach is interesting, there are significant issues for scaling their proposed approach. Their approach required $N{\mathrm{log}}_{2}S$ number of qubits, where ***N*** is the number of vehicles and ***S*** the number of road sections. Even a modest network of 1000 vehicles and 64 road sections, would require 6000 qubits, which would be extremely cost prohibitive.

D-Wave quantum computers (https://www.dwavesys.com/) are essentially a very different quantum computational engine. They rely on the process of “Quantum annealing” to start from a particular system state to that of the final state defined by a Hamiltonian defining the feasible states. As is well known, finding minimum energy states in non-convex Hamiltonians is an NP-hard problem that classical computers take long to solve. The fundamental physical nature of the D-Wave quantum computer makes them feasible to solve an Ising model that is isomorphic to a Quadratic Unconstrained Binary Optimization (QUBO) Problem.

This led to a significant foray into representing some of the transportation problems as a QUBO problem, that could be solved on a D-Wave. These include (a) Travelling Salesman Problem, that has been thoroughly reviewed and evaluated by Warren^9^ (b) Travelling Salesman Problem with Time Windows^10^ (c) Vehicle Routing Problems as well as its variants such as multi-depot capacitated vehicle routing problem (MDCVRP) and its dynamic version^11^ (d) Traffic signal control^12^, and (e) Redistributing and rerouting vehicles for optimal network utilization^13^. It is important to note that Quantum annealing is a meta-heuristic^14^, though it has repeatedly demonstrated to out-perform classical computers to get to more efficient solutions quicker, they do not guarantee optimality until exhausting the search space. Even though quantum computers might outperform classical computers by orders of magnitude, Aaronson^15^ identified NP-Complete problems as one of the limits of Quantum Computers.

Though meta-heuristic approaches are useful, the field of transportation management, policy and planning are subject to scrutiny. This is predominantly because of the safety critical aspects as well as the wide public impact transport interventions have on society. This requires providing structural bounds and assurances on the validity of the solution. This need from a decision support standpoint requires to rely on quantum logic gates.

There has been ground breaking theoretical work that demonstrated quantum algorithms relying on quantum logic gates can provide significant speedups, to name a few: (a) Deutsh-Jozsa algorithm^16^ to determine constant of symmetric output provides exponential speedup (b) Simon’s period finding algorithm^17^ provides exponential speedup (c) Bernstein-Vazirani secret string determination algorithm^18^ provides super-polynomial speedup (d) Grover’s search Algorithm^19^ provides quadratic speedup and (e) Dürr-Høyer algorithm^20^ to find minimum provided polynomial speedup. One of the most celebrated is the Shor's^21^ algorithm, that demonstrated that quantum computers can solve the prime factorization problem exponentially faster than classical computers, having significant implications on cryptography. Though subject to some debate, recently “Quantum Supremacy” was demonstrated on a problem that would take a classical supercomputer 10,000 years to be completed by 53 qubit Sycamore processor in 200 s^22^.

This research contributes by implementing a Quantum Fourier Transform (QFT) to evaluate the frequency domain of the drive cycle. The validity of this method is demonstrated using the IBM quantum simulator (https://quantum-computing.ibm.com/). The QFT algorithm is also implemented on IBM's freely available IBM-Q16 Melbourne, that can be used to create a 15-qubit quantum circuits. The IBM-Q16 does not have a full error correction capability, resulting in noisy outputs. A simple error correction method is proposed that does not compromise the quantum speedups.

## Quantum circuits

Basic information of qubits, Quantum Circuits and Quantum Fourier Transform are comprehensively provided by Nielsen and Chuang^23^. So as to provide completeness of context for broader transportation and traffic researchers, we cover some of the basics. Qubits are the basic unit of quantum information in a quantum circuit. A qubit is represented as a linear superposition of its two orthonormal vectors. These vectors are usually denoted as,

1 $$|0\rangle =\left[\begin{array}{c}1\\ 0\end{array}\right]  \; \text{and} \; |1\rangle =\left[\begin{array}{c}0\\ 1\end{array}\right]$$

Therefore, a qubit can be represented as a linear combination of $|0\rangle$ and $|1\rangle$:

2 $$\varphi =\alpha |0\rangle +\beta |1\rangle  \; \text{Where} \; {\left|\alpha \right|}^{2}+{\left|\beta \right|}^{2}=1$$

It should be noted that $\alpha$ and $\beta$ are complex valued and the corresponding probability amplitudes for $|0\rangle$ and $|1\rangle$. This means that we can measure $|0\rangle$ with a probability of ${\left|\alpha \right|}^{2}$ and $|1\rangle$ with a probability of ${\left|\beta \right|}^{2}$. The concept of “superposition” implies that there is no way to tell which of the two possible states the qubit is in. In fact, the moment we measure a qubit, it collapses to the measured state. As you will see later the probability amplitudes are responsible for quantum “interference”.

A quantum circuit is a sequence of quantum gates that carry out the computation by operating on the qubits. Quantum gates are Unitary operators (U, i.e. $U{U}^{\dagger}=I$), and therefore a quantum circuit is reversible.

Quantum algorithms begin with creating a superposition of qubits that act as an input for the quantum oracle function (${U}_{f}$), which is a quantum version of the classical function ($f$). This process is referred to as “quantum parallelism”, which is widely used as a starting point to build useful quantum algorithms.

### Discrete Fourier transforms

Quantum Fourier transform (QFT) is a quantum implementation of the classical Fourier transform. Before introducing QFT, it is critical to provide a brief overview of a Discrete Fourier Transform (DFT). DFT acts on a complex valued vector $({x}_{0},{x}_{1},\ldots {x}_{N-1})$ to transform it into another complex valued vector $({y}_{0},{y}_{1},\ldots {y}_{N-1})$ according to the formula:

3 $${y}_{k}=\frac{1}{\sqrt{N}}\sum \limits_{j=0}^{N-1}{{x}_{j}e}^{2\pi i\frac{jk}{N}}$$

If ${x}_{j}$ has a period $\tau$, then ${y}_{k}$ is non-zero for $k$ that are multiples of $N/\tau$. Else, it is zero everywhere else. The magnitude of the coefficients of the Fourier basis indicates the amount of that frequency carried in the signal. Therefore, a Fourier transforms a series from the time domain to the frequency domain, making it possible to infer the frequency spectrum. The Fast Fourier Transform is known to have a time complexity of $\mathrm{O}(N\mathrm{log}N)$.

### Quantum Fourier transforms

To employ QFT, we need to define an n-qubit quantum states as inputs, s.t. $N={2}^{n}$. The QFT transforms ${x}_{i}$ to the fourier coefficients ${y}_{i}$. This fourier coefficient ${y}_{i}$ is the same as probability of observing frequency.

4 $$\sum \limits_{i=0}^{N-1}{x}_{i}|i\rangle \stackrel{QFT}{\to }\sum \limits_{i=0}^{N-1}{y}_{i}|i\rangle $$

It is important to note that the probability of measuring state $|i\rangle$ in the standard basis will be ${\left|{y}_{i}\right|}^{2}$. Therefore, applying QFT to a periodic function with period $\tau$ would result in a high likelihood of the measurement of $|k\rangle$, when $k$ is a multiple of $N/\tau$.

A QFT is implemented on a vector of length $N={2}^{n}$, represented by a basis state by $|x\rangle =\left|{x}_{1}\right.\rangle {\otimes } \left|{x}_{2}\right. \rangle {\otimes } \cdots {\otimes }{x}_{n}$ and $x={2}^{n-1}{x}_{n}+\cdots +2{x}_{1}+{x}_{0}$. The coefficient for a frequency from the fourier transform is the same as the probability of observing that frequency. With Eq. (3), we obtain:

5 $$QF{T}_{N}|x\rangle =\frac{1}{\sqrt{N}}\sum \limits_{y=0}^{N-1}{e}^{2\pi i\frac{xy}{{2}^{n}}}|y\rangle $$

Replacing $x$ in Eq. (5) with $x={2}^{n-1}{x}_{n}+\cdots +2{x}_{1}+{x}_{0}$, we obtain:

6 $$QF{T}_{N}|x\rangle =\frac{1}{\sqrt{N}}\sum \limits_{y=0}^{N-1}{e}^{2\pi i\frac{x\sum \limits_{k=1}^{n}{2}^{n-k}{y}_{k}}{{2}^{n}}}|{y}_{1},{y}_{2}\ldots {y}_{n}\rangle $$

7 $$QF{T}_{N}|x\rangle =\frac{1}{\sqrt{N}}\sum \limits_{y=0}^{N-1}\prod_{k=1}^{n}{e}^{2\pi ix{y}_{k}/{2}^{k} }|{y}_{1},{y}_{2}\ldots {y}_{n}\rangle $$

A particular coefficient of $y$ is the probability of observing frequency. After rearranging the sum and products in Eq. (7), we get entangled states:

8 $$QF{T}_{N}|x\rangle =\frac{1}{\sqrt{N}}{\otimes }_{k=1}^{n}(|0\rangle +{e}^{\frac{2\pi ix}{{2}^{k}}}|1\rangle )$$

9 $$QF{T}_{N}|x\rangle ={\otimes }_{k=1}^{n}\frac{1}{\sqrt{2}}(|0\rangle +{e}^{2\pi ix/{2}^{k}}|1\rangle )$$

10 $$QF{T}_{N}|x\rangle ={\otimes }_{k=1}^{n}\frac{1}{\sqrt{2}}(|0\rangle +{e}^{2\pi i\sum \limits_{j=1}^{k}{x}_{n+1-i}/{2}^{i}}|1\rangle )$$

To implement the QFT, we will develop a quantum circuit, which will be explained in the following section.

### Quantum Fourier circuit

**Figure 2:**
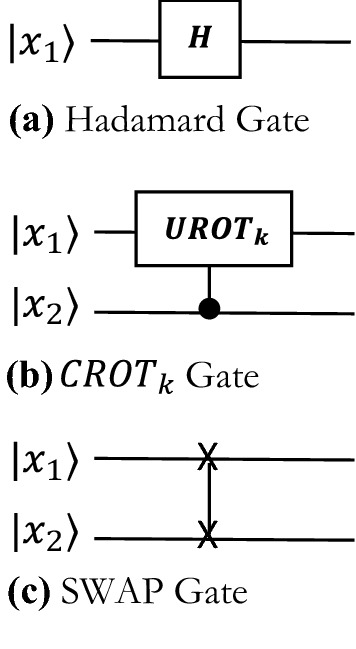
Quantum gates.

A quantum circuit to undertake QFT relies on three types of gates (a) Hadamard Gate (b) $CRO{T}_{k}$ Gate, and (c) Swap Gate. The matrix operations for these three gates are shown in Eqs. (11)–(18), and the circuit representations are shown in Fig. 2.

The Hadamard Gate transforms a qubit ${x}_{k}$

11 $$H=\frac{1}{\sqrt{2}}\left[\begin{array}{cc}1& 1\\ 1& -1\end{array}\right]$$

12 $$H\left|{x}_{k}\right.\rangle =\frac{1}{\sqrt{2}}(|0\rangle +{e}^{\frac{2\pi i{x}_{k}}{2}}|1\rangle )$$

The two-qubit controlled rotation $CRO{T}_{k}$ gate is defined by

13 $$CRO{T}_{k}=\left[\begin{array}{cc}I& 0\\ 0& URO{T}_{k}\end{array}\right]$$

14 $$URO{T}_{k}=\left[\begin{array}{cc}1& 0\\ 0& {e}^{\frac{2\pi i}{{2}^{k}}}\end{array}\right]$$

15 $$CRO{T}_{k}\left|0{x}_{i}\right.\rangle =\left|{0x}_{i}\right.\rangle $$

16 $$CRO{T}_{k}\left|1{x}_{i}\right.\rangle ={e}^{\frac{2\pi i{x}_{i}}{{2}^{k}} }\left|1{x}_{i}\right.\rangle $$

The $SWAP$ gate is defined by

17 $$SWAP=\left[\begin{array}{cccc}1& 0& 0& 0\\ 0& 0& 1& 0\\ 0& 1& 0& 0\\ 0& 0& 0& 1\end{array}\right]$$

18 $$SWAP\left|{x}_{i}{x}_{j}\right.\rangle =\left|{x}_{j}{x}_{i}\right.\rangle $$

**Figure 3:**
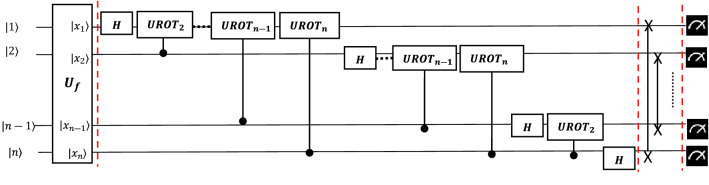
Circuit for a quantum Fourier transform.

A full algorithm follows five steps as shown below to determine the QFT. The corresponding full quantum circuit is shown in Fig. 3. The algorithm starts with an n-qubit input state $|{x}_{1}{x}_{2}\ldots {x}_{n}\rangle$.

1. A Hadamard gate is applied on qubit 1, and the state is transformed from the input state to:
  19 $${H}_{1}\left|{x}_{1}{x}_{2}\ldots {x}_{n}\right.\rangle =\frac{1}{\sqrt{2}}[|0\rangle +{e}^{\frac{2\pi i{x}_{1}}{2}}|1\rangle ] {\otimes } |{x}_{2}{x}_{3}\ldots {x}_{n}\rangle $$
2. Then apply $CRO{T}_{k}$ in series controlled by qubit $k=2\ldots n$. This results in:
  20 $$\frac{1}{\surd 2}[|0\rangle +{e}^{\frac{2\pi i{x}_{1}}{2}+\frac{2\pi i{x}_{2}}{4}\cdots +\frac{2\pi i{x}_{n}}{{2}^{n}}}|1\rangle ] {\otimes } |{x}_{2}{x}_{3}\ldots {x}_{n}\rangle $$
3. Recursively applying Steps (1) and (2) on qubits $i=2..n$.
4. Then apply swap gates to reverse the order of the qubits, to get
  21 $${\otimes }_{k=1}^{n}\frac{1}{\sqrt{2}}(|0\rangle +{e}^{2\pi i\sum \limits_{j=1}^{k}\frac{{x}_{n+1-i}}{{2}^{i}}}|1\rangle )$$
5. Measure the n-qubits.

As can be seen in the circuit shown in Fig. 3, the first qubit would require $n$ gates, i.e., one Hadamard gate and $n-1$ $UROT$ gates. A generic qubit $k$ would require $n-k+1$ gates, followed by $n/2$ swap gates. Therefore, the total number of operations required will be $\left(\frac{n}{2}+\sum \limits_{k=1}^{n}n-k+1\right)=\frac{{n}^{2}}{2}+n$. Hence the complexity of QFT is $O\left({n}^{2}\right)$ or $O{\left(({\ln}N\right)}^{2})$, which is an exponential improvement over a Fast Fourier Transformation. This is a well-known result and is discussed in detail in Nielsen and Chuang^24^.

We demonstrate the application through simulations and implementation on the IBMq16-Melbourne, quantum computer. This is the first application of quantum computing to study traffic dynamics data, particularly in the context of drive cycle evaluation.

## Drive cycle data

In this section, we introduce the drive cycle data used for the simulation. The National Renewable Energy Laboratory (NREL) published drive cycle data that include second-by-second data on vehicle speed and acceleration using a global positioning system (https://www.nrel.gov/transportation/secure-transportation-data/tsdc-drive-cycle-data.html). This acceleration data was used to create a binary variable indicating whether the vehicle was accelerating or decelerating to create a time series comprising of a sequence of binary numbers.

**Figure 4:**
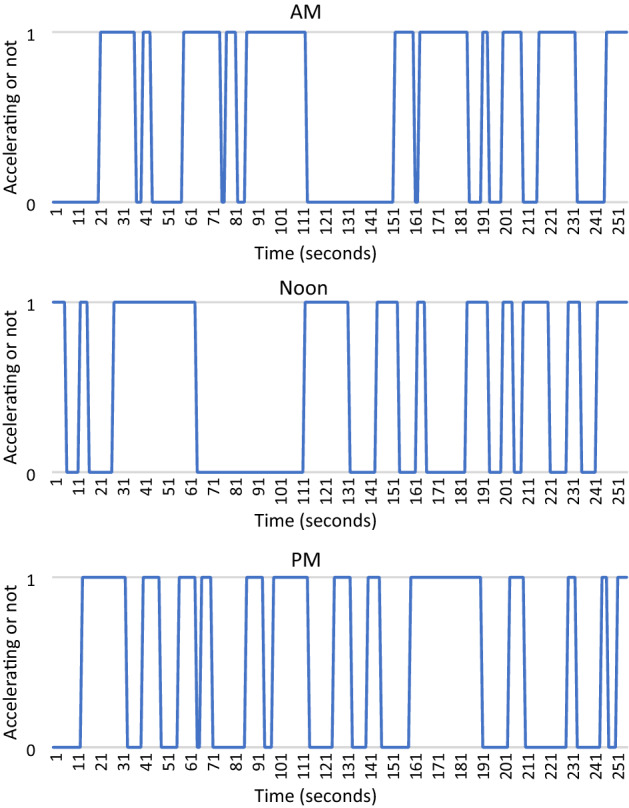
Time series acceleration data.

CALTRANS data from 27th Nov 2012 was used during the following time periods (a) 8:42 A.M.–8:47 A.M. (120–420) (b)13:07 P.M.–13:32 P.M. (15,969–17,512), and (c) 18:37–19:04-(35,770–37,411). The time series data for the acceleration and deceleration during these three time periods are shown in Fig. 4.

Note that a Fourier transforms a series from the time domain to the frequency domain, making it possible to infer the frequency spectrum. In the context of drive cycle estimation, our objective is to apply QFT to transform a series from the acceleration-deceleration time cycles to the frequency domain. In the next section, we will compare the performance of QFT and classical Fourier transform.

## Comparison of quantum Fourier transform with classical Fourier transform

**Figure 5:**
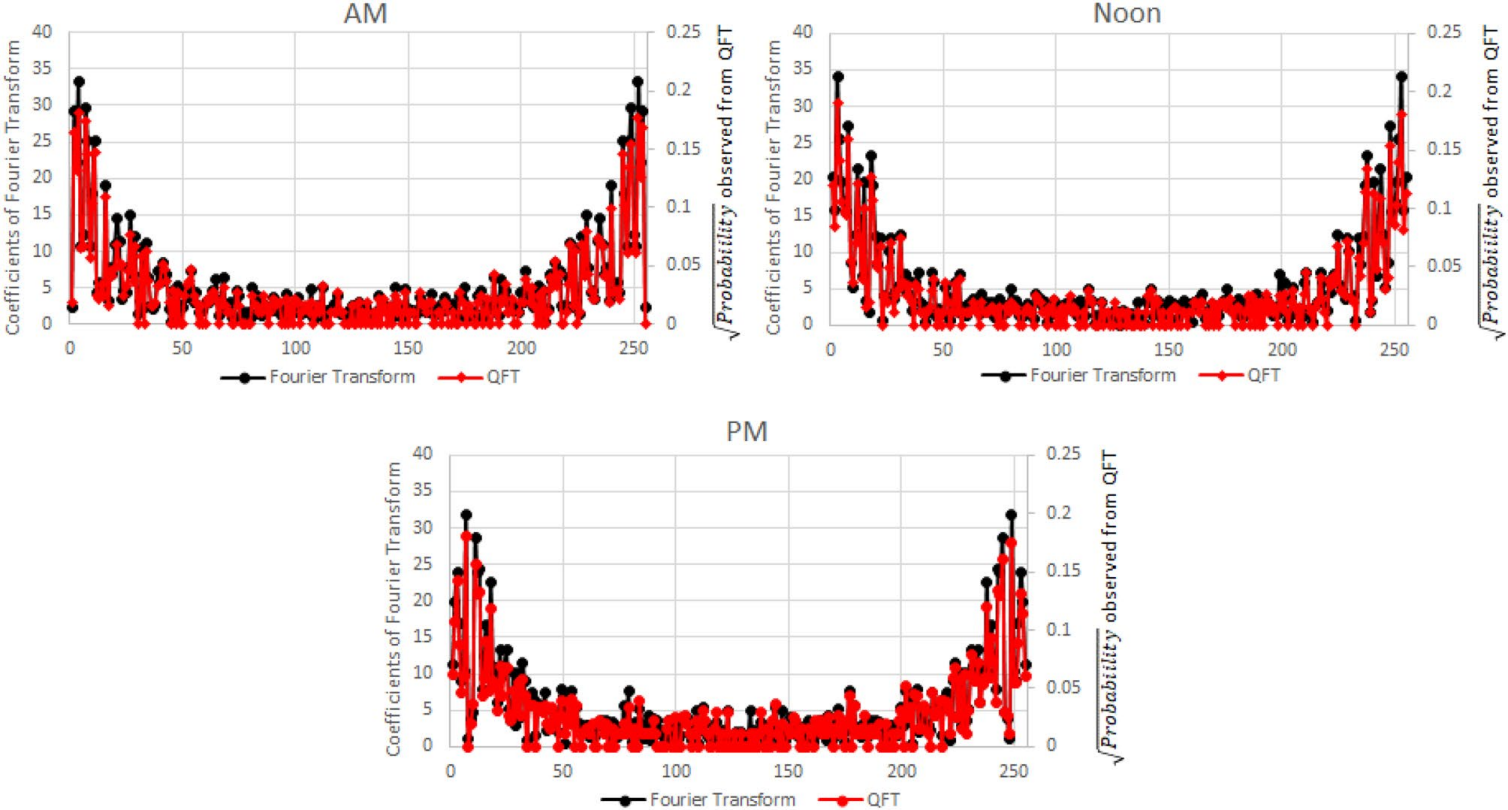
Comparison of the frequency domain based on calculations from QFT and Fourier transform.

**Figure 6:**
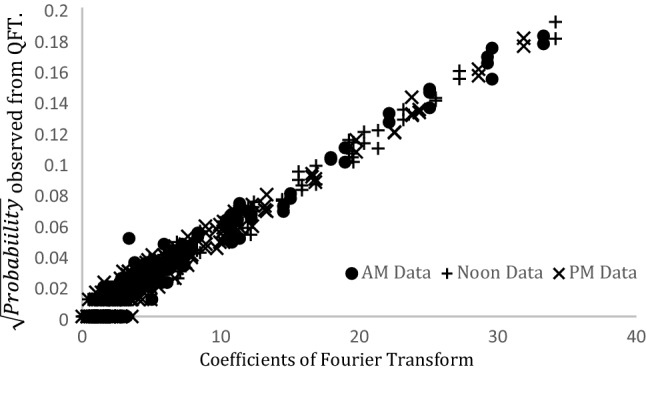
Comparison of the coefficients of the Fourier transform and QFT.

The QFT circuit discussed in “Quantum circuits” section was set up to analyse the frequency domain of the acceleration-deceleration cycles for a 256 s time period. The circuit was run using the IBM Quantum Simulator. The spectral data obtained from the QFT and the classical Fourier transform are in close agreement as seen in Fig. 5. A perfect correlation (correlation coefficient ~ 1) was observed between the square root of the probabilities estimated from the QFT and the coefficients of the Fourier Transform (see Fig. 6).

## QFT using noisy quantum computing

Due to the extreme difficulty in controlling the qubits at low temperatures, maintaining superposition is extremely difficult, resulting in occasional decoherence. Current Quantum Computing Technology tend to be noisy with a small probability of an error in the qubit. Methods to correct these errors are required to develop applications using Noisy Intermediate-Scale Quantum Computing (NISQ)^25^.

The 15 qubit IBM-Q16 Melbourne computer was used for this analysis. This limited the length of the sequence of data on which the QFT can be calculated to be 16. To conduct this analysis, sixteen series of baseline sequences were generated which are referred to as the calibrating dataset and six sequences were randomly generated and are referred to as the evaluation dataset. The 16 sequences were used to were used to calibrate for the underlying error structure and develop the model. The models performance was then evaluated on the 6 sequences.

**Figure 7:**
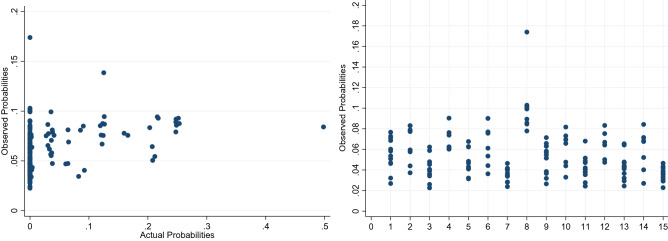
Calibration data. (**a**) Observed vs actual probabilities. (**b**) Spectral analysis of the observed probabilities when actual probabilities is zero.

The probabilities observed from the IBM-Q16 Melbourne were compared with the probabilities generated from the IBM quantum simulator. The comparison between the observed and actual probabilities are shown in Fig. 7a. Though there is a discernible positive correlation of 0.34 at a statistically significance of 95%, there is significant noise that is observed.

A deeper analysis, comparing the observed probabilities with the actual probabilities when those probabilities were zero is shown in Fig. 7b. Other than the consistent bias in the observed probabilities being greater than zero, the magnitude in level of bias varies between the different values in the frequency domain.

As discussed earlier, the QFT implemented on a vector represented by $$x={2}^{n-1}{x}_{n}+\cdots +2{x}_{1}+{x}_{0}$$, results in a probability ($${P}_{y})$$ of observing $$y$$ in the Fourier domain measured as $$y={2}^{n-1}{y}_{n}+\cdots +2{y}_{1}+{y}_{0}$$. In a noisy quantum computer, the transformation in Eq. (4) occurs with errors, assuming that there are independent errors for each $$y$$. Therefore, the observed probabilities $${P}_{y\epsilon }$$ can be written as a function of the actual probabilities $${P}_{y}$$ and the probability of deviating from $$y$$*,* that is $${P}_{y}^{\epsilon }$$ (Eq. 22).

$${P}_{y\epsilon }={P}_{y}\left(1-{P}_{y}^{\epsilon }\right)+{(1-P}_{y}){P}_{y}^{\epsilon }$$

$${P}_{y\epsilon }={P}_{y}+{P}_{y}^{\epsilon }-2{P}_{y}{P}_{y}^{\epsilon }$$

22 $${P}_{y}^{\epsilon }=\frac{{P}_{y\epsilon }-{P}_{y}}{1-2{P}_{y}}$$

**Figure 8:**
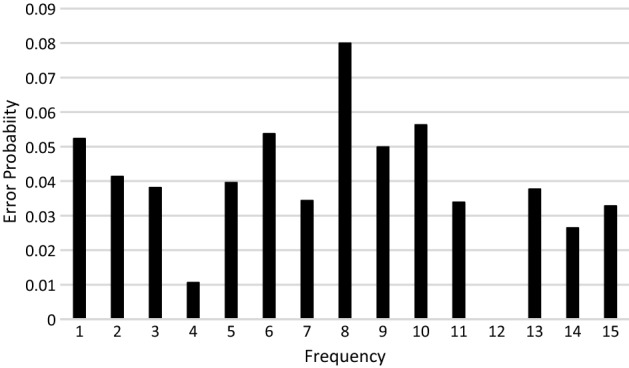
Error probabilities for each frequency value.

We use the calibration dataset to determine the error probability (${P}_{y}^{\epsilon })$ in Eq. (22). Note that in Eq. (22), the actual probabilities ${P}_{y}$ were estimated using the simulator and are therefore known for each $y$. The observed probabilities ${P}_{y\epsilon }$ for each $y$ are obtained from the IBMQ-16 Melbourne quantum computer, which is noisy. Then, given ${P}_{y\epsilon }$ and ${P}_{y}$, ${P}_{y}^{\epsilon }$ can be estimated using Eq. (22) by regressing between the observed and actual probabilities for each $y$ (see Fig. 8).

When a new data stream is analysed using the IBMQ-16 Melbourne quantum computer, and the observed probabilities ${P}_{y\epsilon }$ have noise embedded. To estimate the actual probabilities ($\widehat{{P}_{y}}$) using Eq. (23), we use the calibrated ${P}_{y}^{\epsilon }$ that was determined from the calibration dataset from Eq. (22).

23 $$\widehat{{P}_{y}}=\frac{{P}_{y\epsilon }-{P}_{y}^{\epsilon }}{1-2{P}_{y}^{\epsilon }}$$

**Figure 9:**
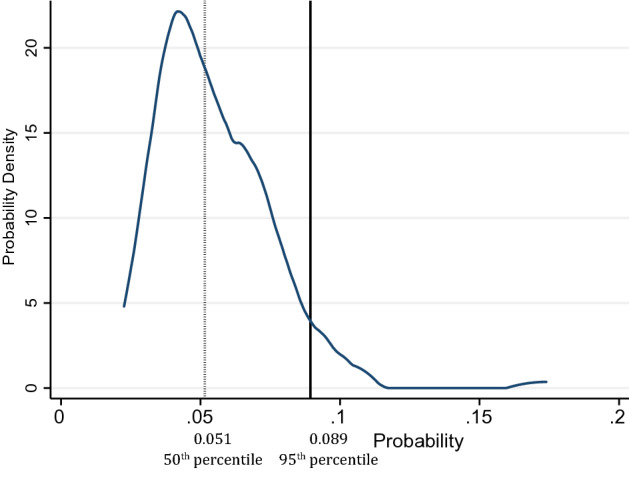
The probability density of the observed probabilities when actual probabilities is zero.

Furthermore, the probability distribution of the observed probabilities when the actual probabilities are zero was found to have a median of 0.051 and the 95th percentile value of 0.089 (see Fig. 9). If the observed probability is less than 0.089, it would not be possible to distinguish if the observed probability appears because the actual probabilities are zero or not. Therefore, in the evaluation, only observed probabilities that are greater than the 95th percentile value of 0.089 are considered. This is denoted by ${P}_{y\epsilon }^{95}$.

**Figure 10:**
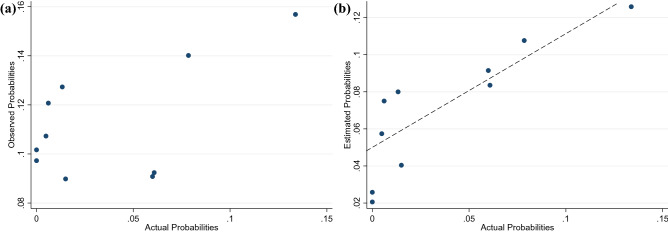
Comparing (**a**) observed and actual probabilities (**b**) estimated and actual probabilities.

**Figure 11:**
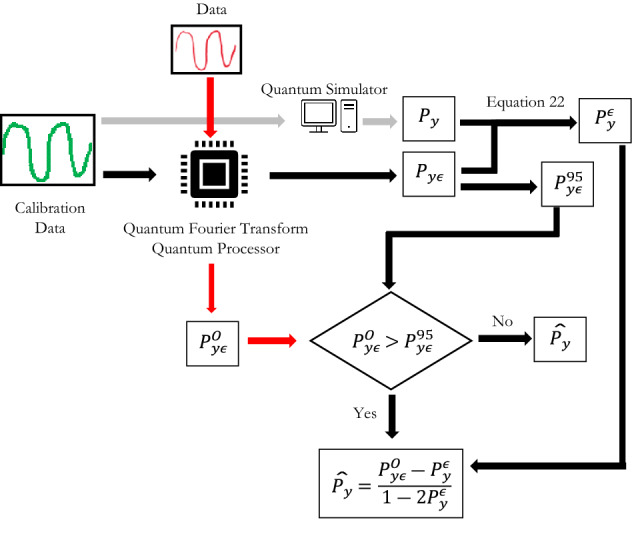
Flow chart of method to estimate probabilities using NISQ.

The estimated probabilities (Fig. 10) align closely with the actual probabilities (correlation of 0.86), as compared to the observed probabilities (correlation of 0.59). Figure 11 summarizes the overall method in a flow chart.

The proposed method using a NISQ based computing system only requires the calibrated error probability (${P}_{y}^{\epsilon }$) and the observed probability (${P}_{y\epsilon }^{O}$), as such it takes a single step to determine the accurate probabilities. Therefore, the computational complexity of post processing to determine the dominant frequency is still $O{\left(({\ln}N\right)}^{2})$. It should be however noted that the dominant frequency needs to have the observed probabilities greater than the threshold (${P}_{y\epsilon }^{95}$). This limits the applications of this method to use cases where the frequencies have observed probabilities greater than the thresholds.

## Conclusion

This paper is one of the first to explore the use of quantum computing for vehicle dynamics and drive cycle analysis. The method particularly relies on the Quantum Fourier Transform (QFT) that is known to be exponentially faster than the Fourier Transform to determine dominant Drive Cycle frequencies.

Using the IBM Quantum Computing Simulators, the study was able to demonstrate that the implementation of the QFT for drive cycle analysis was consistent with the results from the classical Fourier Transform.

Current quantum computers are known to have errors, and in the era of NISQ, it is imperative to develop methods that can achieve quantum speedups despite these errors. The study proposed a simple error correction method to estimate the probabilities consistent with QFT, without compromising the computational complexity. The method was able to reasonably well recover the probabilities.

We are embarking on an exciting frontier of quantum computing that has significant implications on vehicle dynamics, transportation planning and traffic management. These could help with identifying issues quickly and rapidly determining optimal responses, which could in turn help reduce congestion, emissions and improve safety.
